# Lack of Androgen Receptor Expression Selects for Basal-Like Phenotype and Is a Predictor of Poor Clinical Outcome in Non-Metastatic Triple Negative Breast Cancer

**DOI:** 10.3389/fonc.2020.01083

**Published:** 2020-07-28

**Authors:** Nazia Riaz, Romana Idress, Sadia Habib, El-Nasir Lalani

**Affiliations:** ^1^Centre for Regenerative Medicine and Stem Cell Research, Aga Khan University, Karachi, Pakistan; ^2^Section of Breast Diseases, Department of Surgery, Aga Khan University, Karachi, Pakistan; ^3^Department of Pathology and Laboratory Medicine, Aga Khan University, Karachi, Pakistan

**Keywords:** androgen receptor, triple negative breast cancer, basal-like breast cancer, prognosis, cancer stem cells, biomarkers

## Abstract

**Background:** Androgen receptor (AR) has emerged as a significant favorable prognostic indicator in estrogen receptor expressing (ER^+^) breast cancer (BCa); however, its clinical and biological relevance in triple negative breast cancer (TNBC) and association with cancer stem cell (CSC) markers remain ambiguous.

**Methods:** We examined the immunohistochemical expression of AR in a cohort of stage I–III TNBC cases (*n* = 197) with a long-term clinical follow-up data (mean follow-up = 53.6 months). Significance of AR expression was correlated with prognostic biomarkers including cancer stem cell markers (CD44, CD24, and ALDH1), basal markers (CK5, CK14, and nestin), proliferation marker (ki-67), apoptotic marker (Bcl-2), and COX-2. Expression of CK5 and nestin was used for the categorization of TNBC into basal (TN, CK5^+^, and/or nestin^+^) and non-basal (TN, CK5^−^, and/or nestin^−^) phenotypes, and Kaplan–Meier curves were used for estimation of overall survival and breast cancer-specific survival (BCSS).

**Results:** AR expression was observed in 18.8% of non-metastatic TNBC tumors. Expression of AR correlated with lower grade (*P* < 0.001) and conferred a favorable prognostic significance in patients with axillary lymph node metastasis (*P* = 0.005). Lack of AR expression correlated with expression of CSC phenotype (CD44^+^/CD24^−^) (*P* < 0.001), COX-2 (*P* = 0.02), basal markers (CK5: *P* = 0.03), and nestin (*P* = 0.01). Basal-like phenotype (TN, CK5^+^, and/or nestin^+^) correlated with quadruple-negative breast cancer (QNBC) and showed a significant association with adverse prognostic markers including high proliferation index (*P* < 0.001), expression of COX-2 (*P* = 0.009), and CSC phenotype (CD44^+^/CD24^−^: *P* = 0.01). Expression of AR remained an independent prognostic indicator for improved overall survival (*P* = 0.003), whereas basal-like phenotype was associated with an adverse BCSS (*P* = 0.013).

**Conclusions:** Assessment of AR and basal markers identified biologically and clinically distinct subgroups of TNBC. Expression of AR defined a low-risk TNBC subgroup associated with improved overall survival, whereas expression of basal markers (CK5 and nestin) identified a high-risk subgroup associated with adverse BCSS. Integration of immunohistochemical analysis of AR and basal biomarkers to the assessment of TNBC tumors is expected to improve the prognostication of an otherwise heterogeneous disease.

## Introduction

Triple negative breast cancer (TNBC) is an umbrella term encompassing aggressive as well as indolent tumors (1, 2). These heterogeneous tumors comprise 12–17% of all invasive breast cancers (BCa) and are characterized by lack of expression of estrogen receptor (ER), progesterone receptor (PR) and absence of HER2/neu overexpression or gene amplification (3). By this definition of exclusion, these tumors are devoid of existing validated therapeutic targets and chemotherapy remains the main modality of treatment. Considering clinical and biological diversity of these tumors, it is not surprising that clinical outcomes are variable and long-term survival is limited due to aggressive tumor biology and early recurrences (4).

Gene expression profiling has dissected the heterogeneity of TNBC (5) and preclinical data suggest that molecular stratification of these tumors may influence therapeutic options and improve prediction of response to neoadjuvant chemotherapy and prognostication (6). Among these subtypes, AR expressing luminal androgen receptor (LAR) has received substantial attention and has been validated as a stable subtype of TNBC in independent genomic profiling studies. Although being negative for immunohistochemical (IHC) expression of ER, LAR subtype is enriched in hormonally regulated signaling pathways involving steroid synthesis, estrogen/androgen metabolism, and ER-regulated genes (7).

AR is expressed in 10–53% of TNBC tumors, depending on the cutoff used to define positivity (8). Although AR expression has emerged as a promising therapeutic target in LAR subtype of TNBC, its biological role and prognostic significance remains elusive. While AR expressing TNBC tumors are reportedly well-differentiated, slow growing, and are often associated with better survival (9, 10), these observations are contradictory to the gene expression profiling studies and preclinical data where an oncogenic role has been attributed to AR signaling (11). Hence, in view of these discrepant studies, definitive conclusions cannot be drawn about the role of AR in TNBC.

Preclinical studies have shown that ligand-activated AR signaling enriched stem cell pool of CD44^+^/CD24^−^ phenotype in TNBC cell lines, which is effectively abrogated by anti-androgen, enzalutamide (12). However, the prognostic relevance of AR in relation to CSC phenotypes (CD44^+^/CD24^−^ and ALDH1^+^) in TNBC is not known.

Despite the recognition of the molecular subtypes of BCa, current diagnostic methodologies still rely on IHC-based criteria for identification of triple-negative (TN) phenotype, as gene expression profiling assays are not currently recommended for routine diagnosis, risk assessment, or therapy selection (13). Numerous IHC-based surrogate biomarkers have been investigated for subtyping and prognostication, especially for basal-like TNBC, which comprises 9–16% of all BCa cases (14, 15). These aggressive tumors are associated with poor prognosis and are characterized by expression of high-molecular-weight cytokeratins (CKs), which are usually expressed in the basal/myoepithelial layer of the normal breast ducts. The biological relevance of AR and basal biomarkers is being increasingly recognized and evaluation of basal biomarkers in AR^+^ TNBC is evolving as a promising prognostic tool (10, 16, 17).

The aims of the present study were to (1) examine the prevalence of AR expression and basal/non-basal phenotypes in a cohort of TNBC cases; (2) determine the correlation of AR expression and basal/non-basal phenotypes with clinicopathological features, markers associated with CSC phenotype (CD44, CD24, and ALDH1), proliferative index (Ki-67), anti-apoptotic marker (Bcl-2), and COX-2; (3) determine the prognostic significance of AR expression and basal/non-basal phenotypes among patients diagnosed with TNBC.

## Materials and Methods

**Figure 1:**
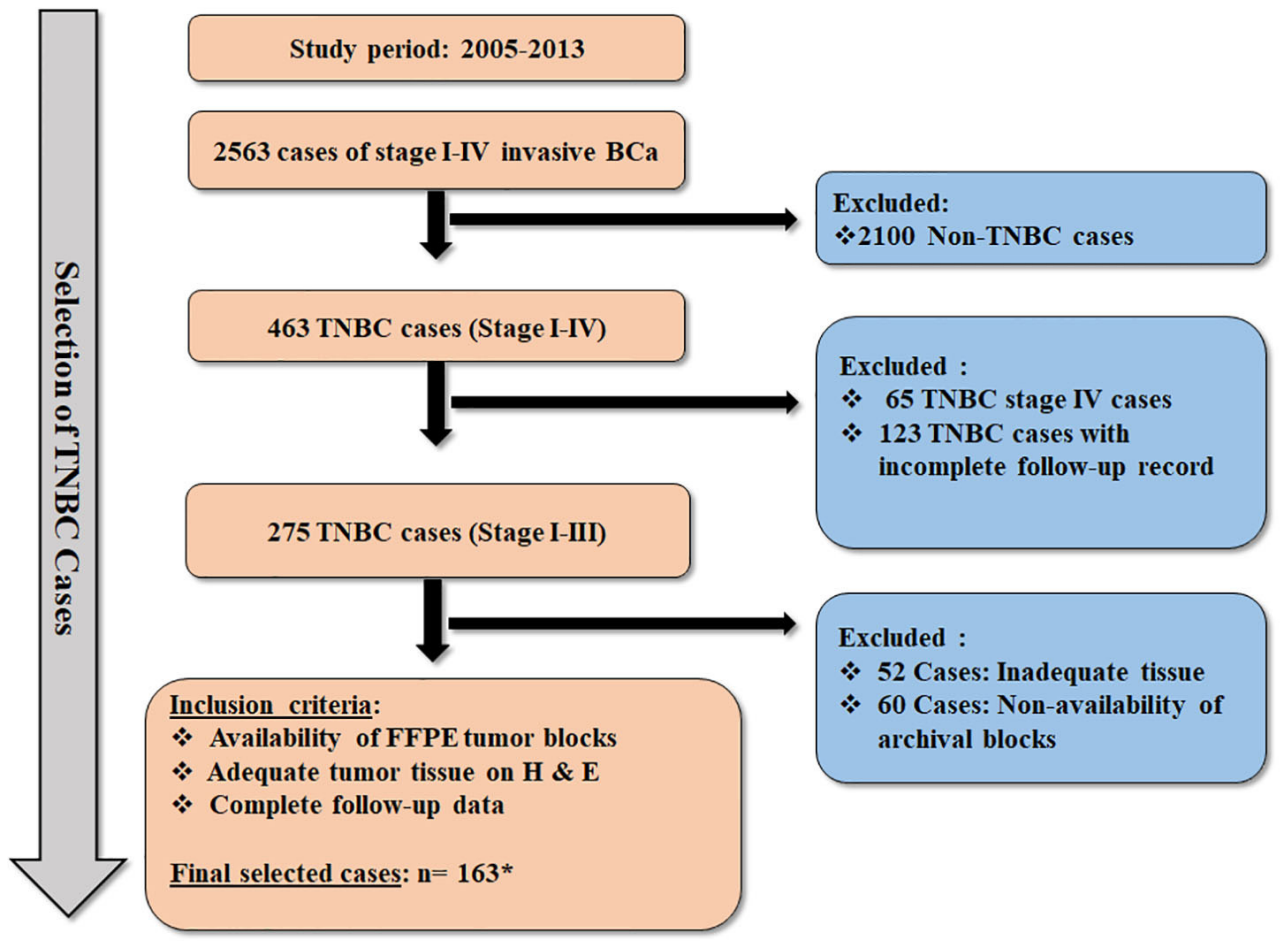
Flow diagram illustrating the selection criteria for the TNBC cases. * Thirty-four cases of TNBC were included from our previous cohort (*n* = 163 + 34 = 197) (18), which was evaluated for expression of AR and CSC markers. However, these 34 cases could not be further assessed for expression of CK5, CK14, nestin, Ki-67, COX2, and Bcl2 due to exhaustion of tumor tissue in FFPE blocks.

Methods are as those described previously (18). Briefly, a retrospective cohort study for the assessment of biomarkers was undertaken at the Aga Khan University Hospital (AKUH), Karachi in accordance with the REMARK guidelines (19). The cases included adult female patients diagnosed with stage I–III TNBC at AKUH who presented and completed their management at the same hospital during the years 2005–2013. Flow diagram illustrating the selection process of TNBC cases is presented in Figure 1.

Medical records were reviewed, and data were collected on structured questionnaire for clinico-pathological characteristics including age, menopausal status, TNM staging, surgical interventions, and systemic therapies administered. Hematoxylin and eosin (H and E)-stained slides of the cases were reviewed by the pathologist who was blinded to the clinical outcome data. Details of tumor type, size, grade, ER/PR, and HER-2/*neu* expression and FISH analysis for HER-2/*neu* gene amplification were retrieved from patients' pathology reports. Follow-up details and outcomes including loco-regional recurrences and deaths were recorded from medical charts.

Study protocol was provided exemption from formal review by the Ethics Review Committee of AKU, Pakistan campus (2517-Pat-ERC-13). All patients had consented for their data and tumor tissues to be used for research. Experimental approach of the study is presented in Figure S1.

### IHC Expression

Formalin-fixed paraffin-embedded (FFPE) archival tissue blocks were retrieved from the Department of Pathology and Laboratory Medicine, AKUH. Appropriate blocks were selected by the pathologist, based on the representative tumor morphology on H and E-stained sections. Serial sections of 5 μm were cut onto poly-L-lysine-coated glass slides (Dako-K8020).

Antibody clones and catalog numbers are presented in Table S1 and details of positive control tissues, antigen retrieval buffer, dilution, and incubation time with the primary antibody are listed in Table S2. Antigen retrieval and staining procedure were performed by using Dako PT-Link and Dako Autostainer, respectively. The addition of chromogen, DAB, and H_2_O_2_ (substrate) resulted in the formation of a brown end-product at the site of the target antigen which was detected by using Dako REAL™ Envision™ Detection System.

All antibodies were optimized on appropriate positive controls (Table S2). Negative control (primary antibody replaced with buffer) and a known positive control were included in each batch. Slides were counterstained with hematoxylin and, once dried, mounted by using DPX mountant.

### Scoring Criteria for IHC Expression

The stained slides were scored by a consultant pathologist, who was blinded to the clinico-pathological data and the outcome of the patients. All slides were evaluated by using an Olympus BX41 microscope at 100× and 200× magnifications. The IHC scoring criteria are detailed in Table S3.

### Statistical Analysis

Statistical analysis was performed using SPSS version 20 software. Descriptive statistics were computed for continuous (mean ± SD) and categorical variables. Duration of follow-up was recorded from the date of diagnosis until death or until the date of last hospital visit at the time of data collection. Loco-regional relapses and deaths were expressed as frequencies. Associations between expression of AR, other markers, and clinico-pathological features were assessed by chi-square test or Fisher exact test, where appropriate, and a *P*-value of <0.05 was considered to be significant. Overall survival (OS) was measured from the date of diagnosis till the date of last follow-up or death. Breast cancer-specific survival (BCSS) was measured from date of diagnosis until death due to BCa. Survival curves were acquired by using Kaplan–Meier methodology and statistical significance between different categories was determined by log rank analysis. Univariable analysis was performed by using Cox proportional hazard model and results were reported as crude hazard ratio. All variables found to have a *P* value of <0.2 in univariable analysis were considered eligible for multivariable analysis and adjusted hazard ratio with 95% confidence intervals was reported using multiple Cox proportional hazard model. Events were defined as deaths and recurrences attributed to BCa.

## Results

### Patient and Tumor Characteristics

A total of 2,563 cases of stage I–IV primary invasive BCa were diagnosed between 2005 and 2013 at the Department of Pathology and Laboratory Medicine, AKUH. Based on the archival pathology reports, 463 cases of TNBC were identified as ER^−^, PR^−^, and HER-2/*neu*^−^, which accounted for 18% of TNBC cases (463/2,563). A total of 197 stage I–III cases of TNBC were considered evaluable, fulfilling the inclusion criteria (Figure 1).

Clinico-pathological characteristics of the 197 cases are presented in Table 1. The median age of patients was 49.5 years (±12.3) and just over half of the patients were post-menopausal (56.9%) at the time of diagnosis. Most of the TNBC cases were diagnosed with stage II disease (67.5%) with grade III tumors (82.7%) and 61% cases had axillary lymph node (LN) negative disease. Surgical intervention is comprised of mastectomy in 69.5% of cases. All patients received standard systemic therapy in adjuvant (67.5%) or neo-adjuvant (32.5%) setting and 71% of the cases also received radiation therapy.

**Table 1:** Clinico-pathological features of TNBC cases (*n* = 197).

**Clinico-pathological features**	**Frequency (%)**
**Mean age**	49.5 (SD: 12.3)
≤40	48 (24.4)
>40	149 (75.6)
**Menopausal status**	
Pre-menopausal	85 (43.1)
Post-menopausal	112 (56.9)
**Tumor size**	
≤2 cm	59 (29.9)
>2 cm	138 (70.1)
**Tumor grade**	
I	2 (1)
II	32 (16.2)
III	163 (82.7)
**Axillary lymph node status**	
N0	120 (60.9)
N1	41 (20.8)
N2	23 (11.7)
N3	13 (6.6)
**TNM stage**	
I	12 (6.1)
II	133 (67.5)
III	52 (26.4)
**Surgery**	
Breast Conservation	60 (30.5)
Mastectomy	137 (69.5)
**Systemic therapy**	
Adjuvant	133 (67.5)
Neo-adjuvant	64 (32.5)
**Radiation therapy**	
Yes	140 (71.1)
No	57 (28.9)

### Frequency of Expression of Various Biomarkers

**Figure 2:**
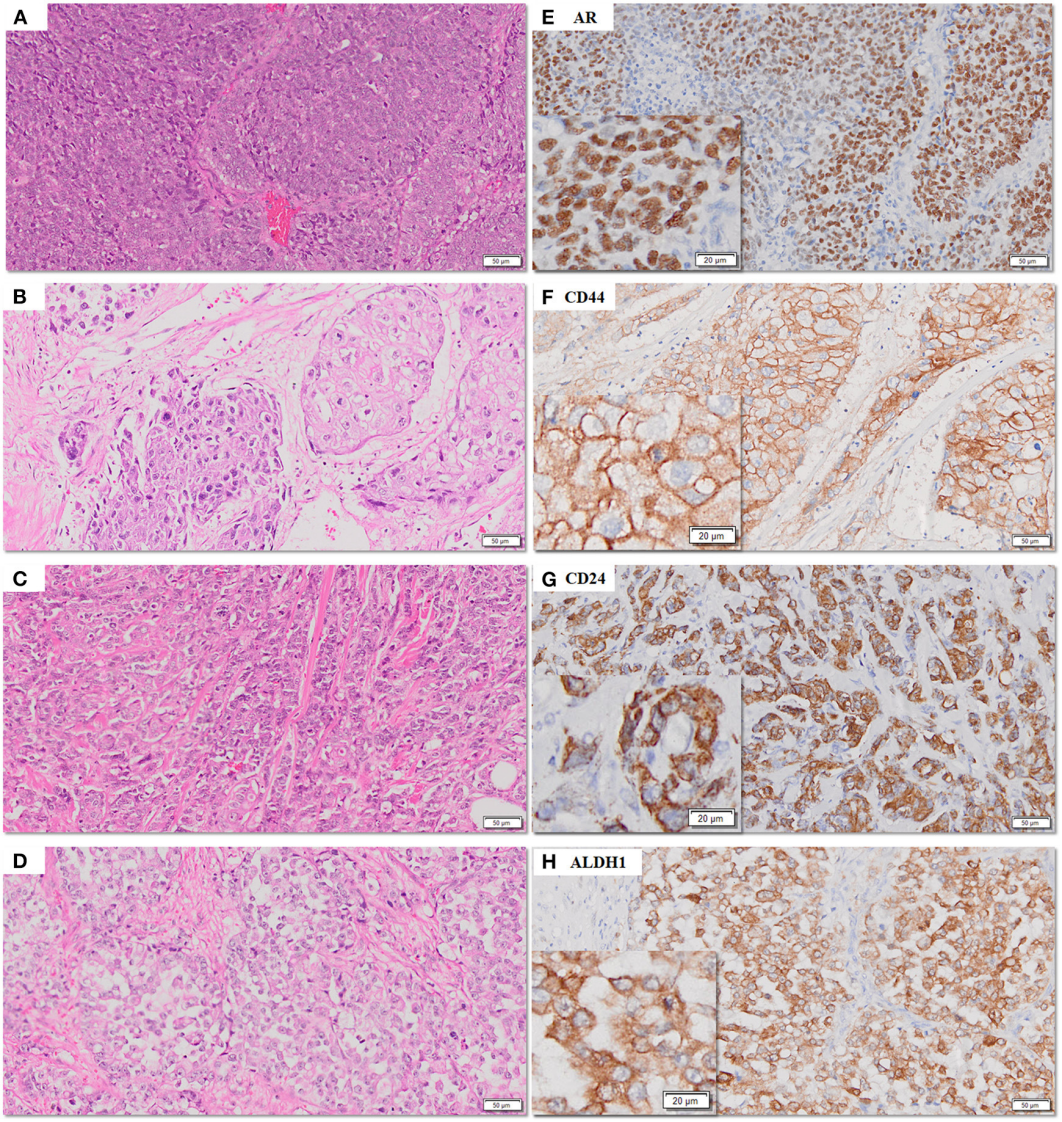
**(A–H)** Representative photomicrographs for expression of AR, CD44, CD24, and ALDH1 in TNBC sections. AR (nuclear), CD44 (membranous), CD24 (cytoplasmic), and ALDH1 (cytoplasmic) in TNBC **(E–H)** with corresponding hematoxylin and eosin stained sections **(A–D)** [scale bar, 50 μm (original magnification, 10×); inserts: scale bar, 20 μm (original magnification, 20×)].

**Figure 3:**
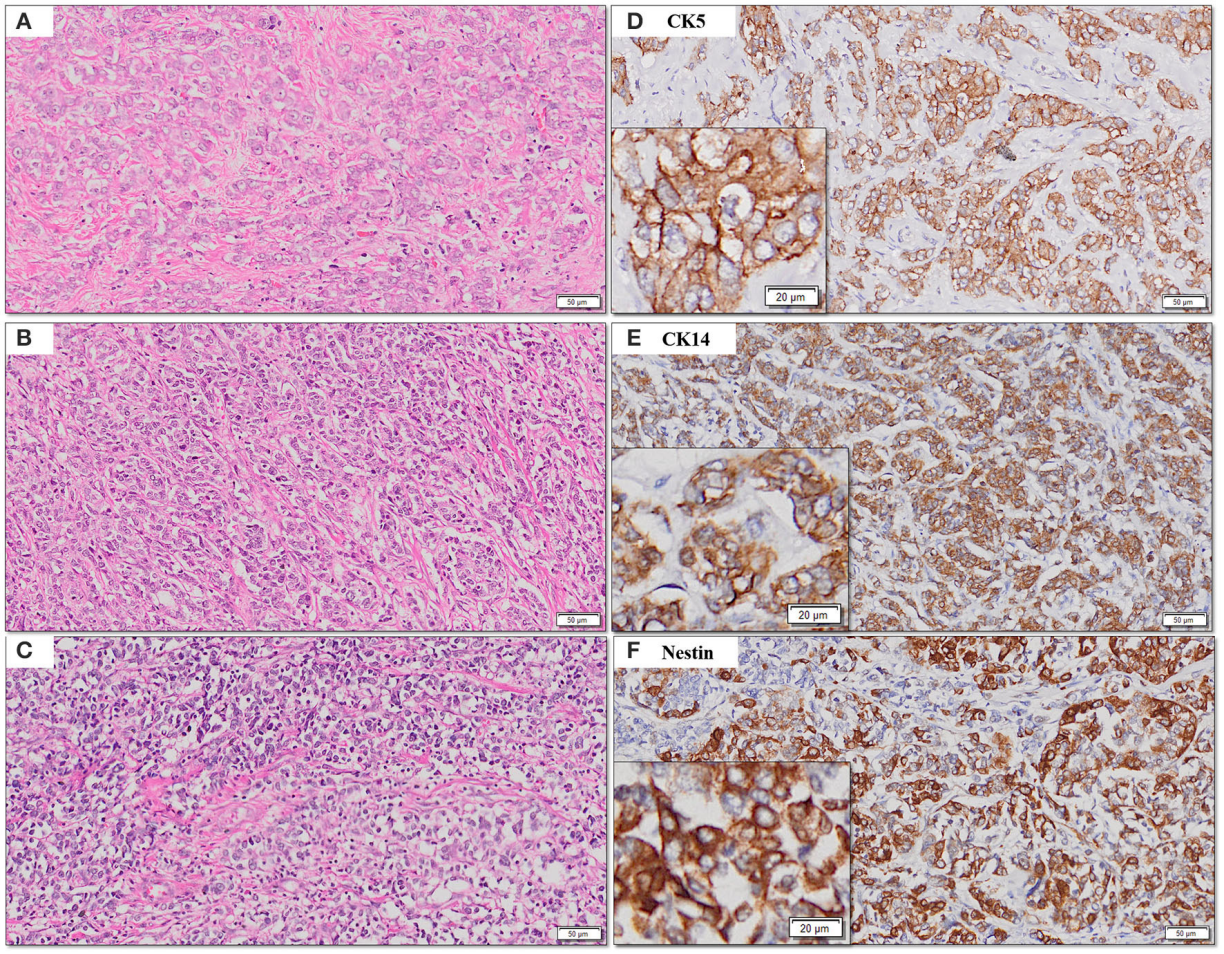
**(A–F)** Representative photomicrographs for cytoplasmic expression of CK5, CK14, and nestin in TNBC **(D–F)** with corresponding hematoxylin and eosin stained sections **(A–C)** [scale bar, 50 μm (original magnification, 10×); inserts: scale bar, 20 μm (original magnification, 20×)].

**Figure 4:**
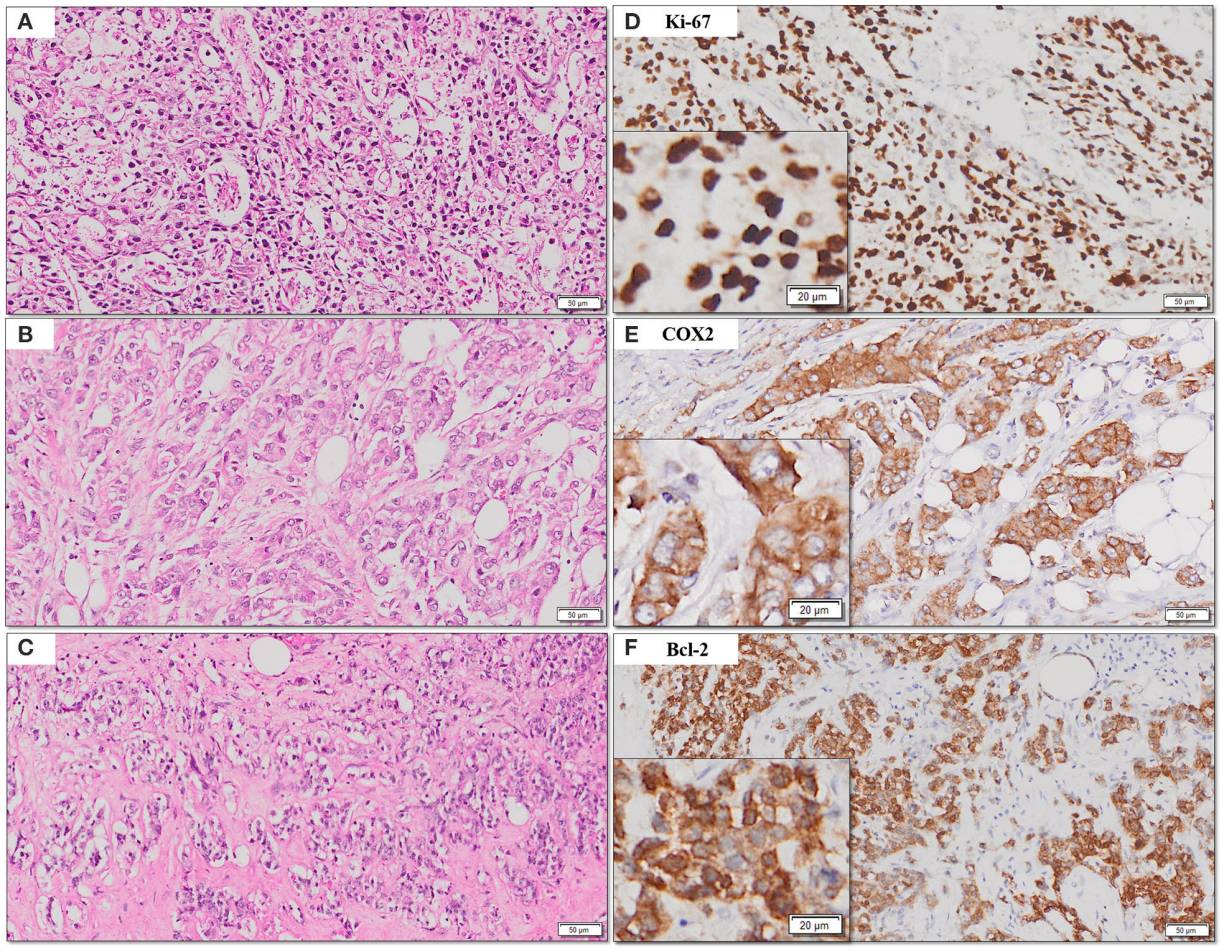
**(A–F)** Representative photomicrographs for expression of Ki-67, COX2, and Bcl2. Nuclear expression of Ki-67 **(B)**; cytoplasmic expression of COX2 and Bcl-2 **(C,D)** with corresponding hematoxylin and eosin stained sections **(A–C)** [scale bar, 50 μm (original magnification, 10×); inserts: scale bar, 20 μm (original magnification, 20×)].

The frequency of expression of various biomarkers is summarized in Table 2 and photomicrographs of representative H and E sections and expression of markers are shown in Figures 2A–H, 3A–F, 4A–F.

**Table 2:** Frequency of expression of various biomarkers.

**Markers**	**Frequency of expression (%)**
***n*** **=** **197**	
**AR expression (Allred criteria)**	
Negative (≤2)	160 (81.2)
Positive (>2)	37 (18.8)
**CD24 expression**	
Negative	132 (67)
Positive	65 (33)
**CD44 expression**	
Negative	139 (70.6)
Positive	58 (29.4)
**ALDH1 expression**	
Negative	171 (86.8)
Positive	26 (13.2)
***n*** **=** 163^*^	
**CK5 expression**	
Negative	85 (52.1)
Positive	78 (47.9)
**Nestin expression**	
Negative	100 (61.3)
Positive	63 (38.7)
**CK14 expression**	
Negative	88 (54)
Positive	75 (46)
**Ki-67 expression**	
≤25%	111 (68.1)
>25%	52 (31.9)
**Bcl-2 expression**	
Negative	120 (73.6)
Positive	43 (26.4)
**COX2 expression**	
Negative	41 (25.2)
Intermediate	85 (52.1)
High	37 (22.7)

* *34 tumor samples could not be assessed for the expression of CK5, Nestin, CK14, Ki-67, Bcl2, and COX-2 due to exhaustion of the tumor tissue on FFPE blocks*.

### Factors Associated With AR Expression

In accordance with the Allred scoring criteria, a score of >2 for AR expression was considered positive and was observed in 18.8% (37/197) of the TNBC cases. The relationship between AR expression and clinico-pathological characteristics is summarized in Table 3. AR expression inversely correlated with grade III tumors (*P* < 0.001). Significant correlations were not observed with other clinical or pathological features.

**Table 3:** Clinico-pathological features stratified by AR expression (*n* = 197).

**Parameter**	QNBC^*^	**AR^**+**^TNBC**	***P-*value**
	***n* = 160 (81.2%)**	***n* = 37 (18.8%)**	
**Age at diagnosis (years)**			
≤40	37 (77.1)	11 (22.9)	0.39
>40	123 (82.6)	26 (17.4)	
**Menopausal status**			
Pre	70 (82.4)	15 (17.6)	0.72
Post	90 (80.4)	22 (19.6)	
**Tumor size**			
≤2 cm	46 (88.1)	7 (11.9)	0.10
>2 cm	108 (78.3)	30 (21.7)	
**Tumor grade**			
I	0	2 (100)	**<0.001**
II	21 (65.6)	11 (34.4)	
III	139 (85.3)	24 (14.7)	
**Lymph node status**			
N0	88 (73.3)	32 (26.7)	0.64
N1	29 (70.7)	12 (29.3)	
N2	15 (69.2)	8 (34.8)	
N3	11 (84.6)	2 (15.4)	
**TNM stage**			
I	11 (91.7)	1 (8.3)	0.33
II	110 (82.7)	23 (17.3)	
III	39 (75)	13 (25)	
**Surgery**			
BCT	50 (83.3)	10 (16.7)	0.61
Mastectomy	110 (80.3)	27 (19.7)	
**Systemic therapy**			
Adjuvant	108 (81.2)	25 (18.8)	0.99
Neoadjuvant	52 (81.2)	12 (18.8)	
**Radiation therapy**			
Yes	117 (83.6)	23 (16.4)	0.18
No	43 (75.4)	14 (24.6)	

* *QNBC, Quadruple-negative breast cancer. Bold values indicate statistical significance (p < 0.05)*.

The association of AR expression and biomarkers is presented in Table 4. Among the panel of markers used as IHC surrogates for basal-like phenotype, expression of CK5 and nestin inversely correlated with AR expression (*P* < 0.05), and a similar trend of marginal significance was also observed for CK14 (*P* = 0.05).

**Table 4:** Expression of biomarkers stratified by AR expression.

**Marker**	**QNBC**	**AR^**+**^TNBC**	***P*-value**
***n*** **=** **163**			
**CK5 expression**			
Negative	69 (89.2)	16 (18.8)	**0.03**
Positive	72 (92.3)	6 (7.7)	
**CK14 expression**			
Negative	72 (81.8)	16 (18.2)	0.05
Positive	69 (92)	6 (8)	
**Bcl-2 expression**			
Negative	100 (83.3)	20 (16.7)	**0.04**
Positive	41 (95.3)	2 (4.7)	
**COX2 expression**			
Negative	39 (92.7)	3 (7.3)	**0.02**
Intermediate	76 (89.4)	9 (10.6)	
High	27 (73)	10 (27)	
**Nestin expression**			
Negative	81 (81)	19 (19)	**0.01**
Positive	60 (95.2)	3 (4.8)	
**Ki-67 expression**			
≤25%	96 (86.5)	15 (13.5	0.99
>25%	45 (86.5)	7 (13.5)	
***n*** **=** **197**			
**CD24 expression**			
Negative	119 (90.2)	13(9.9)	**<0.001**
Positive	41(63.1)	24 (36.9)	
**CD44 expression**			
Negative	117 (84.2)	22 (15.8)	0.1
Positive	43 (74.1)	15 (25.9)	
**CD44/CD24 phenotypes**			
CD44^−^/CD24^−^	85 (91.4)	8 (8.6)	**<0.001**
CD44^+^/CD24^+^	8 (44.4)	10 (55.6)	
CD44^−^/CD24^+^	32 (69.6)	14 (30.4)	
CD44^+^/CD24^−^	35 (87.5)	5 (12.5)	
**ALDH1 expression**			
Negative	133 (87.5)	19 (12.5)	0.16
Positive	8 (72.7)	3 (27.3)	

*Bold values indicate statistical significance (p < 0.05)*.

AR expression was infrequently observed in tumors with Bcl-2 (*P* = 0.04) and intermediate to high expression of COX-2 (*P* = 0.02). Among the CSC markers, the CD44^+^/CD24^−^ phenotype correlated with lack of expression of AR (*P* < 0.001). Significant associations were not observed with CD44, ALDH1, and Ki-67 (*P* > 0.05).

### IHC Surrogate Markers for Categorization of Basal-Like vs. Non-basal-Like TNBC

In addition to the negative expression of ER, PR, and HER2/neu, several biomarkers have been evaluated for identification of basal-like BCa. Among these, IHC expression of nestin and CK5 have demonstrated accurate identification of basal-like subtype BCa. Both are intermediate filaments and are expressed in abundance in the basal/myoepithelial layer of the normal breast ducts (20). Hence, in this study, TNBC cases were stratified into basal and non-basal TNBC based on the following criteria:

a. Basal-like TNBC: (ER^−^, PR^−^, HER-2/*neu*^−^, CK5^+^, and/or nestin^+^)

b. Non-basal TNBC (ER^−^, PR^−^, HER-2/*neu*^−^, CK5^−^, and/or nestin^−^).

The correlation of various markers with basal and non-basal subtypes is presented in Table 5. Basal-like phenotype was significantly associated with high Ki-67 proliferation index (*P* = 0.005), intermediate/high expression of COX-2 (*P* = 0.009), and CD44^+^/CD24^−^ phenotype (*P* = 0.01), while the non-basal TNBC phenotype was associated with expression of AR (*P* < 0.001) and CD24 (*P* = 0.01).

**Table 5:** Expression of biomarkers stratified by basal and non-basal phenotypes.

**Marker**	**Non-basal TNBC**	**Basal-like TNBC**	***P-*value**
	***n* = 56 (34.3%)**	***n* = 107 (65.6%)**	
**AR expression**			
Negative	41 (29.1)	100 (70.9)	**<0.001**
Positive	15 (68.2)	7 (31.8)	
**Ki-67 expression**			
< /=25%	46 (41.4)	65 (58.6)	**<0.005**
>25%	10 (19.2)	42 (80.8)	
**Bcl-2 expression**			
Negative	48 (38.3)	74 (61.7)	0.07
Positive	10 (23.3)	33 (76.7)	
**COX2 expression**			
Negative	20 (48.8)	21 (51.2)	**0.009**
Intermediate	20 (23.5)	65 (76.5)	
High	16 (43.2)	21 (56.8)	
**CD24 expression**			
Negative	32 (28.3)	81 (71.7)	**0.01**
Positive	24 (48)	26 (52)	
**CD44 expression**			
Negative	50 (37.6)	83 (62.4)	0.06
Positive	6 (20)	24 (80)	
**CD44/CD24 phenotype**			
CD44^−^/CD24^−^	27 (30)	63 (70)	**0.01**
CD44^+^/CD24^+^	1 (16.7)	5 (83.3)	
CD44^−^/CD24^+^	23 (53.5)	20 (46.5)	
CD44^+^/CD24^−^	5 (20.5)	19 (79.2)	
**ALDH1 expression**			
Negative	52 (34.2)	100 (65.8)	0.88
Positive	4 (36.4)	7 (63.6)	

*Bold values indicate statistical significance (p < 0.05)*.

### Prognostic Significance of TNBC Cohort Stratified by Expression of AR and Basal vs. Non-basal Subtypes

Mean OS was 9.2 years (±0.41). A total of 52 events were recorded in the entire cohort with 13 recurrences (local/systemic) and 39 deaths attributed to BCa. The OS and BCSS rates were 84% and 88% for the AR^+^ TNBC group, respectively, whereas patients in the QNBC (TNBC/AR^−^) group experienced lower overall and BCSS rates of 51% and 72%, respectively.

**Figure 5:**
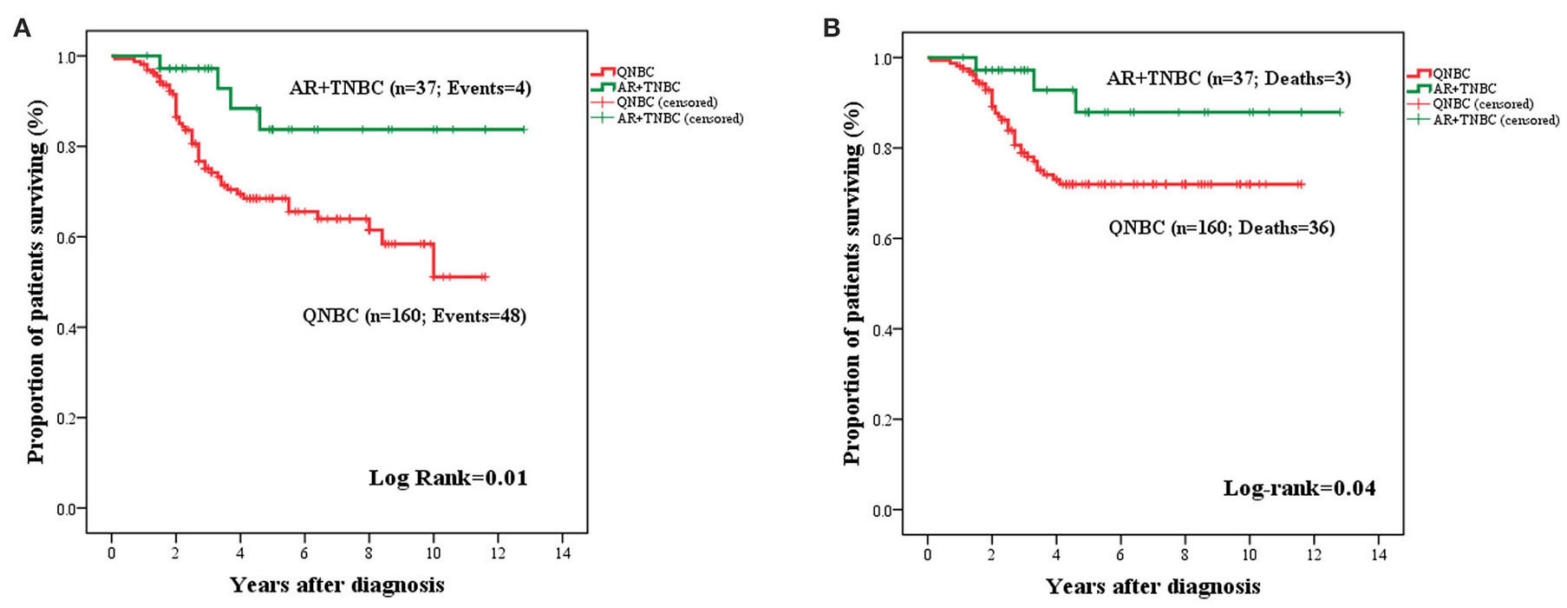
Kaplan–Meier curves for OS **(A)** and BCSS **(B)** in TNBC stratified by AR expression (*n* = 197). AR^+^TNBC cases experienced an improved OS **(A)** and BCSS **(B)** as compared to cases with QNBC.

Lack of AR expression in tumors with QNBC phenotype was associated with significantly adverse OS (mean: 8.11 years ± 0.04; 95% CI: 7.32–8.90 vs. mean: 11.28 years ± 0.70; 95% CI: 9.90–12.65: *P* = 0.01) and BCSS (mean: 9.01 ± 0.37; 95% CI: 8.29–9.74 vs. mean: 11.66 ± 0.62; 95% CI: 10.44–12.88; *P* = 0.04) (Figure 5).

**Figure 6:**
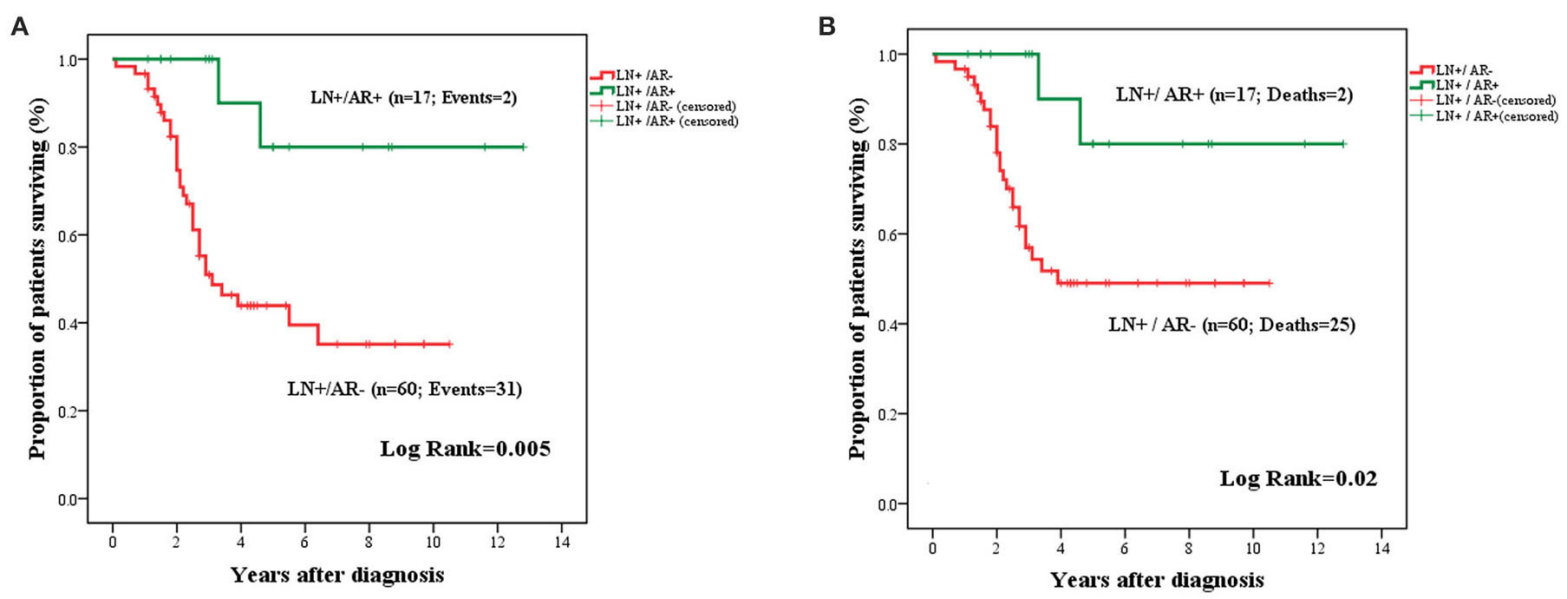
Kaplan–Meier curves for OS **(A)** and BCSS **(B)** in LN^+^ TNBC cases stratified by AR expression. Expression of AR in the primary tumor was associated with improved OS **(A)** and BCSS **(B)** among patients with LN metastasis.

The prognostic significance of AR expression was evaluated in a subgroup of patients with LN-positive (*n* = 77) and LN-negative disease status (*n* = 120). AR expression conferred significantly improved OS in LN^+^ patients (mean: 11.03 years ± 1.12; 95% CI: 8.82–13.23 vs. 5.42 years ± 0.58; 95% CI: 4.28–6.57; *P* = 0.005) (Figure 6). However, this survival advantage was not observed in LN^−^ cases (Figure S2).

**Figure 7:**
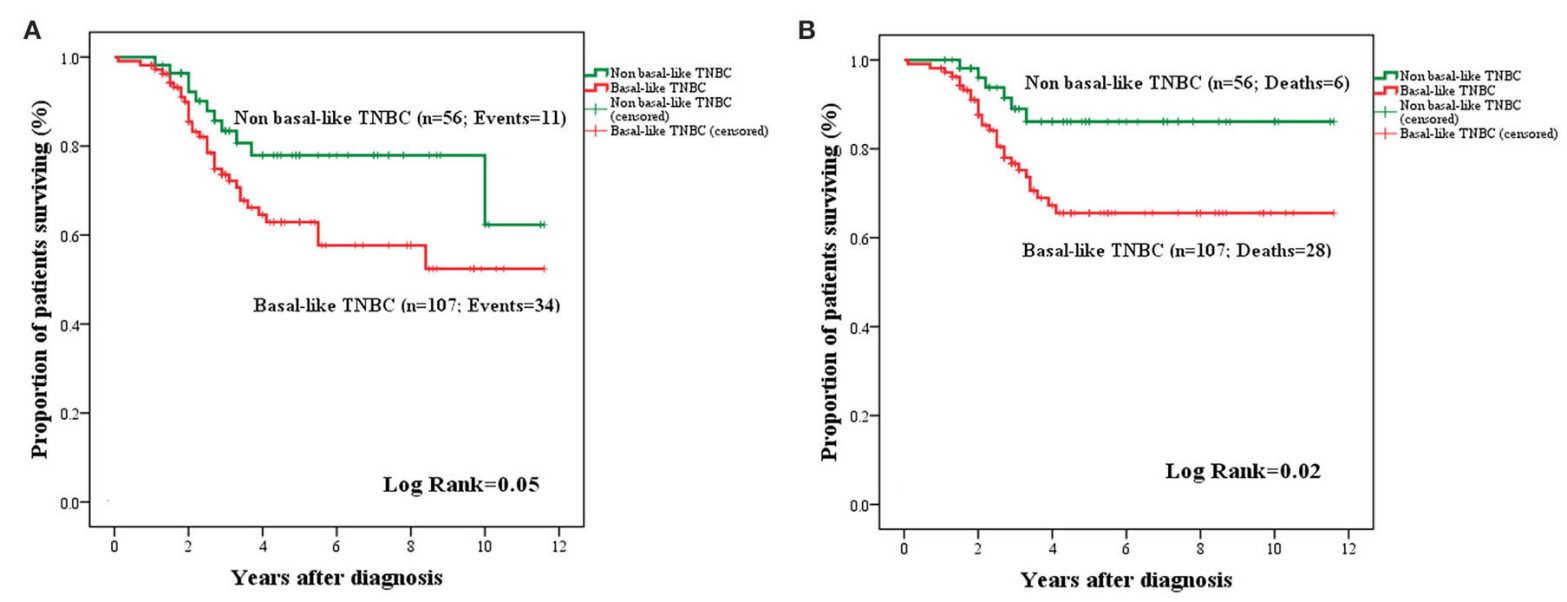
Kaplan–Meier curves for OS **(A)** and BCSS **(B)** in basal and non-basal-like TNBC. Basal-like BCa cases were associated with a trend toward adverse OS **(A)** and significantly adverse BCSS **(B)** compared to non-basal like TNBC.

The OS and BCSS for basal (TN, CK5^+^, and/or nestin^+^) and non-basal (TN, CK5^−^, and/or nestin^−^) subgroups are presented in Figure 7. An adverse OS (mean: 7.71 ± 0.52 years; 95% CI: 6.68–8.73 vs. mean: 9.33 ± 0.59; 95% CI: 8.17–10.49; *P* = 0.05) and BCSS (mean: 8.45 ± 0.49; 95% CI: 7.48–9.42 vs. mean: 10.33 ± 0.48; 95% CI: 9.39–11.28; *P* = 0.02) were attributed to tumors with basal-like phenotype.

Univariable and multivariable analysis for OS and BCSS are presented in Tables 6, 7, respectively. AR expression was associated with significantly longer OS conferring 79% decreased risk for an adverse event compared to QNBC (adjusted HR: 0.202; 95% CI: 0.071–0.573; *P* = 0.003). Basal-like TNBC phenotype (TN, CK5^+^, and /or nestin^+^) was associated with significantly shorter BCSS (adjusted HR: 3.060; 95% CI: 1.261–7.425; *P* = 0.013), conferring a three times higher risk for BCa-related deaths compared to tumors with non-basal TNBC phenotype. Advanced stage and metastasis to the axillary LN were associated with adverse outcome for both OS and BCSS. However, expression of CSC phenotypes (CD24, CD44^+^, CD44^+^/CD24^−^, and ALDH1^+^) was not contributory to the survival prognostication in TNBC patients.

**Table 6:** Univariable analysis for OS and BCSS.

**Variable**	***N***	**Univariable analysis**			
		**OS**		**BCSS**	
		**Crude hazard ratio (95% CI)**	***P*-value**	**Crude hazard ratio (95% CI)**	***P*-value**
**Age (years)**					
≤40	48	1	0.75	1	0.98
>40	149	1.111 (0.571–2.64)		1.009 (0.479-2.128)	
**Tumor size (cm)**					
≤2	59	1	0.08	1	0.06
>2	138	1.777 (0.913–3.458)		2.140 (0.944-4.848)	
**Tumor grade**					
I and II	34	1	**0.02**	1	0.22
III	163	2.733 (1.081–6.910)		1.781 (0.696-4.561)	
**Axillary LN**					
Negative	120	1	**<0.001**	1	**<0.001**
Positive	77	(1.985–6.159)		4.374 (2.21–8.643)	
**TNM stage**					
I and II	145	1	**0.001**	1	**0.001**
III	52	2.525 (1.463–4.359)		3.036 (1.620–5.690)	
**Surgery**					
BCT	60	1	0.27	1	0.12
Mastectomy	137	1.417 (0.756–2.655)		1.814 (0.834–3.946)	
**Systemic therapy**					
Adjuvant	133	1	0.06	1	0.12
NAC	64	1.633 (0.956–2.893)		1.640 (0.870–3.091)	
**Radiation therapy**					
No	57	1	**0.04**	1	**0.02**
Yes	140	2.063 (1.006–4.234)		2.886 (1.129–7.379)	
**AR expression**					
Negative	160	1	**0.01**	1	0.05
Positive	37	0.312 (0.112–0.867)		0.327 (0.101–1.061)	
**CD24 expression**					
Negative	132	1	0.99	1	0.88
Positive	65	1.004 (0.566–1.779)		0.950 (0.488–1.850)	
**CD44 expression**					
Negative	139	1	0.51	1	0.48
Positive	58	1.213 (0.679–2.166)		1.268 (0.061–2.469)	
**CD44/CD24 phenotype**					
CD44^−^/CD24^−^	93	1	0.74	1	0.84
CD44+/CD24+	18	0.941 (0.357–2.483)		1.018 (0.344–3.008)	
CD44^−^/CD24+	46	1.093 (0.544–2.199)		0.904 (0.393–2.081)	
CD44+/CD24^−^	40	1.445 (0.718–2.910)		1.353 (0.607–3.017)	
**ALDH1 expression**					
Negative	171	1	0.73	1	0.09
Positive	26	0.872 (0.392–1.938)		0.317 (0.076–1.317)	
**TNBC subtype**					
Non-basal	107	1	0.05	1	**0.02**
Basal	56	1.910 (0.965–3.780)		2.763 (1.144–6.675)	

*Bold values indicate statistical significance (p < 0.05)*.

**Table 7:** Multivariable analysis for OS and BCSS.

**Variable**	***n***	**Multivariable analysis**			
		**Overall survival**		**BCSS**	
		**Adjusted hazard ratio (95% CI)**	***P*-value**	**Adjusted hazard ratio (95% CI)**	***P*-value**
**Axillary nodal status (*****n*** **=** **197)**					
Negative	120	1	**<0.001**	1	**<0.001**
Positive	77	3.146 (1.733–5.711)		4.822 (2.219–10.479)	
**TNM stage (*****n*** **=** **197)**					
I/II	145	1	**0.006**	1	**0.001**
III	52	2.245 (1.256–4.013)		3.163 (1.591–6.288)	
**AR expression (*****n*** **=** **197)**					
Negative	160	1	**0.003**	1	0.41
Positive	37	0.202 (0.071–0.573)		0.534 (0.119–2.397)	
**TNBC subtype (*****n*** **=** **163)**					
Non-basal	107	1	0.11	1	**0.013**
Basal-like	56	1.777 (0.868–3.641)		3.061 (1.261–7.427)	

*Bold values indicate statistical significance (p < 0.05)*.

## Discussion

To the best of our knowledge, this is the first study to report the prognostic significance of AR expression in a TNBC cohort from Pakistan where age-standardized incidence rates are highest among the Asian countries. In addition, an alarming increase in BCa incidence has been predicted over the next 10 years (21). Hence, this genetic and ethnic diversity merits evaluating the expression of AR and other biomarkers in high-risk, less-studied populations.

In this study, we have shown that AR was expressed in 18.8% of operable stage I–III TNBC treated with standard cytotoxic chemotherapy and radiation therapy. In addition, expression of AR was significantly associated with better OS (*P* = 0.01) and BCSS (*P* = 0.04) and emerged as an independent prognostic indicator for OS in multivariable analysis (*P* = 0.003).

Contrary to other studies (22, 23), we did not find a significant correlation between AR expression and favorable clinico-pathological tumor characteristics such as small tumor size and early stage. These discrepant observations may be attributed to the predominant aggressive tumor biology of our TNBC cohort where only 6% of the patient population was diagnosed with stage I disease and about 40% of the patients had axillary LN metastasis at initial diagnosis. The plausible factors associated with aggressive tumor biology among Pakistani women compared to Western, Asian, and African cohorts require further genetic and epidemiological studies (24).

We have demonstrated that loss of AR in QNBC is significantly associated with expression of CK5, CK14, and nestin (*P* < 0.05). This observation is in concordance with previous studies where QNBC correlated with basal like phenotype associated with disease progression and poor prognosis (25). We further observed significant positive expression of COX-2 and Bcl-2 in QNBC. These observations provide some insight into possible mechanisms governing the aggressive biology of QNBC. Firstly, preclinical data have shown that COX-2 overexpression in the transgenic mouse model is sufficient to induce malignant mammary tumors (26). Furthermore, autocrine and paracrine signaling mediated by COX-2 and its principal metabolic product, prostaglandin E2 (PGE2), are critical for motility, invasion, metastasis, and evasion of apoptosis (27). One of the mechanisms proposed for COX-2-induced deregulation of apoptosis is by increasing Bcl-2 expression in cancer cells (28), an effect that is abrogated by COX-2 inhibitors (29). Conversely, AR+TNBC was found to have low expression of Bcl-2 and COX-2. Preclinical studies have shown that the Bcl-2 promoter has ARE binding sites and that ligand activation of AR in prostate cancer cell lines directly represses Bcl-2 transcription (30) and COX2 expression via modulation of NF-κB signaling (31).

Our subgroup analysis has shown that AR expression of the primary tumor conferred improved OS and BCSS among patients with LN metastasis, whereas AR expression did not contribute to survival advantage in patients without LN metastasis. This is a confounding observation that needs validation and elucidation in future studies. Nevertheless, evidence from preclinical studies supports the idea that ligand activation of AR represses the transcription of long non-coding RNA, ARNILA, which in turn induces upregulation of mir204 inhibiting SOX4-mediated epithelial–mesenchymal transition (EMT) and metastasis (32).

Previous studies have shown that CSC phenotypes (CD44^+^/CD24^−^ and ALDH1^+^) are abundantly expressed in TNBC and are associated with poor outcome (33). However, clinical studies investigating the potential prognostic implications of AR and CSC marker expression in TNBC are lacking. Only two previous studies have examined the expression of AR and ALDH1 in TNBCs. However, they did not report the relevance of AR and ALDH1 with clinical outcome (34, 35). This may be due to challenges associated with collection of long-term clinical follow-up data from developing countries. Our results indicate that independent expression of CSC phenotypes (CD44^+^/CD24^−^ and ALDH1^+^) did not influence the survival outcomes in the TNBC cohort. Moderate sample size limited our ability to perform subgroup analysis to ascertain the prognostic significance of AR with CSC phenotypes.

There is compelling clinical and preclinical evidence supporting the dualistic role of AR in TNBC; however, to date, lack of consensus prevails. Clinical studies investigating the prognostic relevance of AR in TNBC have yielded inconsistent results. Some studies, like ours, have demonstrated that AR^+^ TNBC cases were associated with a favorable outcome (36, 37), while other studies either failed to report any significant effect of AR expression on survival (38) or reported an aggressive tumor biology, lower pathological complete response rates, and dismal prognosis (39, 40).

Preclinical studies exploring mechanistic effects of AR signaling have also produced conflicting results. On one hand, a tumor suppressive role has been attributed to AR signaling, whereby treatment of AR^+^ TNBC cell lines with AR agonists inhibited CSC phenotype (41), decreased EMT by upregulating AR inducible micro RNAs (42), and inhibited growth of xenograft tumors by modulating paracrine signaling (43). On the other hand, there is evidence to support that AR transcription facilitated oncogenesis by activating PIK3 kinase pathway as mutations of PIK3CA kinase and amplification of PIK3 kinase locus are frequently encountered in both AR^+^ TNBC tumors and cell lines (44). The pro-survival role of AR in TNBC is further supported by *in vitro* studie*s* where AR signaling yielded a proliferative effect and a CSC-like state in AR^+^ TNBC cell lines, thus facilitating tumor stemness (12).

In view of these pleiotropic roles, the prognostic significance and effective targeting of AR in TNBC remains an ongoing debate. It is not surprising that AR agonists, antagonists, and inhibitors of androgen synthesis are being investigated as potential therapeutic agents in TNBC, although the field is predominated by inhibitors of AR signaling, with low to modest clinical benefit rates. A noticeable observation emanating from these clinical trials is the inconsistency in defining AR positivity for patient selection (45, 46).

In addition to the variations in IHC protocols and scoring criteria, the divergent roles of AR signaling are possibly influenced by several other important factors: Firstly, ethnic diversity of the patient cohorts may potentially influence the incidence and prognostic relevance of AR expression in TNBC. This was highlighted in a recent multi-institutional study comprising of 1,407 TNBC patients from four continents where AR expression and prognostic relevance demonstrated population-specific variations (47). Whether these differences are influenced by CAG repeat polymorphism of AR and levels of endogenous circulating androgens is an exciting, yet unexplored field (48, 49). Secondly, mutations of AR may potentially alter the downstream AR intracellular signaling pathways as well as sensitivity to ligand binding. These mutations may not be recapitulated in IHC studies on tumor specimens (50). Thirdly, epigenetic factors including histone modifications and methylation of AR promoter may also account for heterogeneous expression of AR in TNBC and may variably influence the AR signaling and outcomes (51). Fourthly, similar to prostate cancer, it has been shown that expression of AR splice variant such as AR-V7 lacking all or a portion of ligand binding domain results in constitutively active AR and regulates a transcriptional program distinct from that of full-length AR (52). Lastly, AR activity is dependent not only on AR but also on the expression of AR synthesizing enzymes in tumor tissues. It has been shown that concordant expression of enzymes involved in AR synthesis and AR may have an anti-proliferative effect as opposed to their discordant expression (53).

TNBC has been recognized as a clinically and molecularly distinct entity for more than two decades; however, the current clinical management of these heterogeneous tumors is still largely governed by the “ER^−^, PR^−^ and HER-2/*neu*^−^ status” as determined by IHC and/or FISH. Although gene expression profiling is the gold standard to identify basal like BCa, its utility in diagnostic labs is challenging. Hence, several IHC surrogate biomarkers have been validated against the gene expression platform ranging from structural proteins (cytokeratins and claudins) to those involved in signal transduction and apoptosis (54). In this study, we utilized positive expression of nestin and CK5 in addition to TN phenotype as a surrogate for basal like phenotype. Both biomarkers have been validated against microarray-based gene expression profile as promising surrogates for identification of basal-like BCa (20).

Although retrospective in nature, the strength of our study is a detailed analysis of a well-annotated cohort of TNBC patients treated with standard chemotherapy and radiotherapy with availability of long-term follow-up data. The association of AR expression in tumors of patients with CSC phenotypes could not be assessed due to inadequate sample size and warrants examination in larger cohorts.

## Conclusions

The analysis presented in this study underscores the importance of AR expression in the primary tumor as a robust and independent biomarker in non-metastatic TNBC and provides prognostic information in patients with LN metastasis. Given the strong correlation with prognosis, we recommend integration of IHC analysis of AR and basal biomarkers to the assessment of TNBC tumors for improving the prognostication of an otherwise heterogeneous disease.

## Data Availability Statement

The datasets generated for this study are available on request to the corresponding author.

## Ethics Statement

The studies involving human participants were reviewed and approved by Ethics Review Committee, Aga Khan University, Karachi. The patients included in the study provided written informed consent prior to surgical intervention for the use of the tumor tissues and data for research.

## Author Contributions

E-NL conceived the project. E-NL and NR designed the experiments, reviewed the data and drafted the manuscript. NR conducted the experiments, performed statistical analysis and interpreted the data. RI and NR undertook pathological review of the slides with interpretation. SH assisted with some experimental work. All authors contributed to the article and approved the submitted version.

## Conflict of Interest

The authors declare that the research was conducted in the absence of any commercial or financial relationships that could be construed as a potential conflict of interest.

## Supplementary Material

Figure S1 Experimental approach of the study.

Figure S2 Kaplan–Meier curves for OS **(A)** and BCSS **(B)** in LN^−^ TNBC cases stratified by AR expression. There was no significant difference in OS **(A)** and BCSS **(B)** among patients without LN metastasis.

Table S1 Antibody clones, catalogue numbers, and manufacturer's details.

Table S2 Details of the positive control tissues, retrieval buffer, dilution, and incubation time of the antibodies.

Table S3 Scoring criteria for IHC expression.
