# Supplementary figures and images for: Lack of Androgen Receptor Expression Selects for Basal-Like Phenotype and Is a Predictor of Poor Clinical Outcome in Non-Metastatic Triple Negative Breast Cancer
Source: Front Oncol. 2020 Jul 28;10:1083. doi: 10.3389/fonc.2020.01083 (PMC7399239; doi:10.3389/fonc.2020.01083)

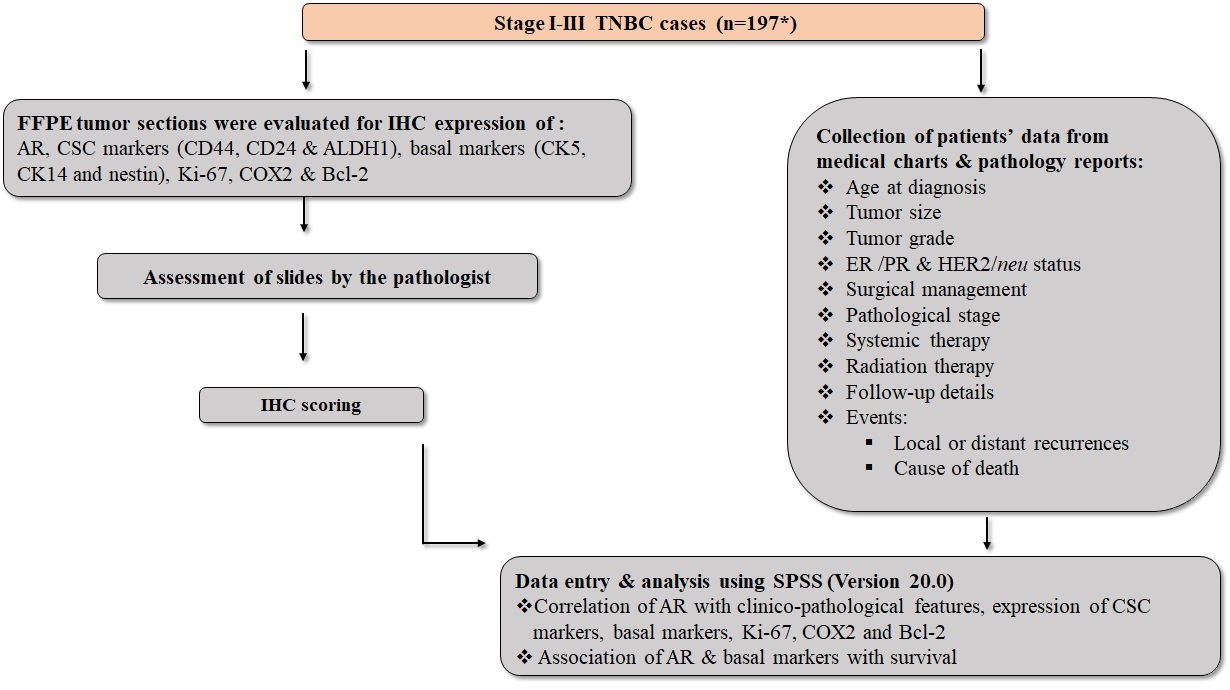

Supplement: Figure S1 — Experimental approach of the study. [file Image_1.TIF]

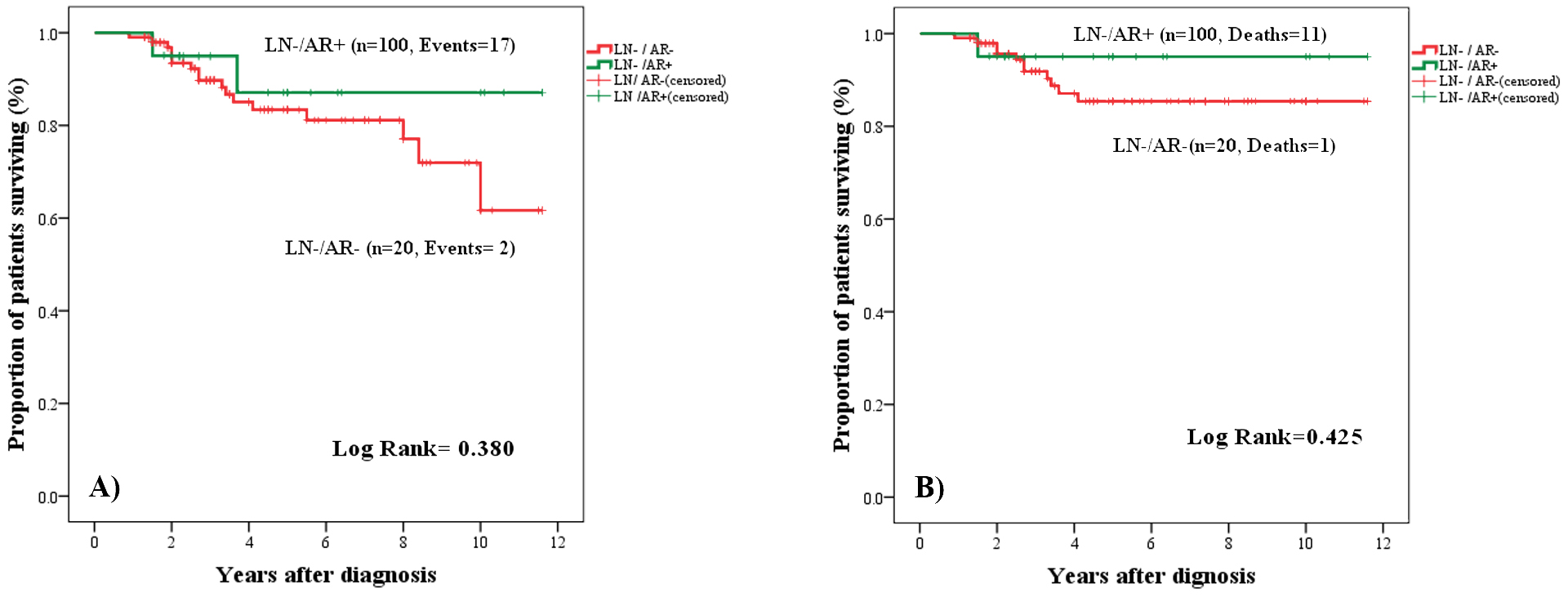

Supplement: Figure S2 — Kaplan–Meier curves for OS (A) and BCSS (B) in LN− TNBC cases stratified by AR expression. There was no significant difference in OS (A) and BCSS (B) among patients without LN metastasis. [file Image_2.JPEG]
