# Supplementary material for: Lack of Androgen Receptor Expression Selects for Basal-Like Phenotype and Is a Predictor of Poor Clinical Outcome in Non-Metastatic Triple Negative Breast Cancer
Source: Front Oncol. 2020 Jul 28;10:1083. doi: 10.3389/fonc.2020.01083 (PMC7399239; doi:10.3389/fonc.2020.01083)
Supplement: Table S1 — Antibody clones, catalogue numbers, and manufacturer's details. [file Data_Sheet_1.docx]

Supplementary Material

# Supplementary Tables

**Table S1| Antibody clones, catalogue numbers and manufacturer’s details**

| **Antibody** | **Clone** | **Catalogue #** | **Species** | **Company** |
| --- | --- | --- | --- | --- |
| **ALDH1** | 44 | 611195 | Mouse | BD Biosciences^™^ |
| **AR** | AR441 | M3562 | Mouse | Dako \| Agilent |
| **Bcl2** | 124 | MO887 | Mouse | Dako \| Agilent |
| **CD44** | DF1485 | M7082 | Mouse | Dako \| Agilent |
| **CD24** | SN3b | MS1279 | Mouse | ThermoFisher Scientific |
| **CK5** | XM26 | NCL-L-CK5 | Mouse | Leica Biosystems |
| **CK14** | LL002 | NCL-L-LL002 | Mouse | Leica Biosystems |
| **COX2** | CX-294 | M3617 | Mouse | Dako \| Agilent |
| **Ki-67** | MIB-1 | M7240 | Mouse | Dako \| Agilent |
| **Nestin** | 10c2 | sc-23927 | Mouse | Santa Cruz Biotechnology, Inc |

**Table S2| Details of the positive control tissues, retrieval buffer, dilution and incubation time of the antibodies**

| **Antibody** | **Positive control tissues** | **Target Retrieval Solution^*^** | **Dilution** | **Incubation time (min)** |
| --- | --- | --- | --- | --- |
| **ALDH1** | IDC^**^ /liver | Low pH | 1:750 | 60 |
| **AR** | Luminal cells of BPH^***^ | Low pH | 1:100 | 60 |
| **Bcl2** | Tonsil | High pH | 1:250 | 60 |
| **CD44** | IDC | Low pH | 1:100 | 60 |
| **CD24** | Normal epithelium of small and large bowel /colon Ca | High pH | 1:100 | 60 |
| **CK5** | Myoepithelial cells of normal breast ducts | Low pH | 1:100 | 60 |
| **CD14** | Myoepithelial cells of normal breast ducts | Low pH | 1:100 | 60 |
| **COX2** | Basal cells in ductal hyperplasia | High pH | 1:100 | 60 |
| **Ki-67** | IDC nuclei | High pH | 1:100 | 60 |
| **Nestin** | Myoepithelial cells of normal breast ducts/IDC | Low pH | 1:100 | 60 |

^*Agilent |Dako; **IDC= Infiltrating ductal carcinoma; *** BPH=benign prostatic hyperplasia^

**Table S3| Scoring criteria for IHC expression**

| **Marker** | **Expression pattern** | **Scoring criteria** | **Reference** |
| --- | --- | --- | --- |
| **ALDH1** | Cytoplasmic | Percentage (***P)*** and intensity (***I***) of cytoplasmic expression was recorded:  ***Intensity (I) of Expression***: 0=negative; 1=weak; 2=moderate; 3=strong  ***ALDH1 Score (S) = P x I***  For statistical analysis:  Score =0 (Negative)  Score >0 (Positive) | [1] |
| **AR** | Nuclear | **Allred Score:**  ***Proportion Score (PS):***  0=0; 1=1/100; 2=1/10; 3=1/3; 4=2/3; 5= 1  ***Intensity Score (IS):***  0= Negative; 1= Weak; 2 = Intermediate; 4= Strong  ***Allred Score= PS + IS***  Negative: ≤ 2  Weak: 3-4  Intermediate: 5-6  Strong: 7-8 | [2] |
| **Bcl-2** | Cytoplasmic | Positive: ≥10% cytoplasmic expression  Negative: <10% cytoplasmic expression | [3] |
| **CD44** | Membranous | 0=no expression; 1=1-10% positive tumor cells; 2=11-50%; 3=51-75%; 4=76-100% | [4] |
| **CD24** | Cytoplasmic | 0=no expression; 1=1-10% positive tumor cells; 2=11-50%; 3=51-75%; 4=76-100% | [4] |
| **CK5** | Cytoplasmic | Positive expression: Any proportion of cytoplasmic expression in tumor cells | [5] |
| **CK14** | Cytoplasmic | Positive expression: Any proportion of cytoplasmic expression in tumor cells | [5] |
| **COX2** | Cytoplasmic | Weighted score was computed based upon the percentage of tumor positivity and intensity as follows: | [6] |
|  |  | ***Score Percent Staining (%)***  0 <5  1 5-25  2 26-50  3 51-75  4 >75  ***Score Intensity***  0 Absent  1+ Weak  2+ Medium  3+ Strong    ***Weighted Score = % of tumor cell positivity x intensity:***  Negative: 0-4  Intermediate: 5-8  High: 9-12 |  |
| **Ki-67** | Nuclear | At least 500 tumor cells were counted, and a threshold of >25% nuclear expression was considered to be positive, irrespective of the staining intensity. | [7] |
| **Nestin** | Cytoplasmic /membranous | Expression was defined as either positive (at least 3 clearly positive tumor cells) or negative. | [8] |

1. Ginestier, C., et al., *ALDH1 is a marker of normal and malignant human mammary stem cells and a predictor of poor clinical outcome.* Cell Stem Cell, 2007. **1**(5): p. 555-67.

2. Allred, D.C., et al., *Prognostic and predictive factors in breast cancer by immunohistochemical analysis.* Mod Pathol, 1998. **11**(2): p. 155-68.

3. Callagy, G.M., et al., *Bcl-2 is a prognostic marker in breast cancer independently of the Nottingham Prognostic Index.* Clin Cancer Res, 2006. **12**(8): p. 2468-75.

4. Honeth, G., et al., *The CD44+/CD24- phenotype is enriched in basal-like breast tumors.* Breast Cancer Res, 2008. **10**(3): p. R53.

5. Banerjee, S., et al., *Basal-like breast carcinomas: clinical outcome and response to chemotherapy.* J Clin Pathol, 2006. **59**(7): p. 729-35.

6. Half, E., et al., *Cyclooxygenase-2 expression in human breast cancers and adjacent ductal carcinoma in situ.* Cancer Res, 2002. **62**(6): p. 1676-81.

7. Petrelli, F., et al., *Prognostic value of different cut-off levels of Ki-67 in breast cancer: a systematic review and meta-analysis of 64,196 patients.* Breast Cancer Res Treat, 2015. **153**(3): p. 477-91.

8. Kruger, K., et al., *Expression of Nestin associates with BRCA1 mutations, a basal-like phenotype and aggressive breast cancer.* Sci Rep, 2017. **7**(1): p. 1089.
